# Chemical Purity by Dielectric Cryometry

**DOI:** 10.6028/jres.067A.060

**Published:** 1963-12-01

**Authors:** Gaylon S. Ross, Lois J. Frolen

## Abstract

A consideration of the deficiencies in standard methods used for the cryometric determination of purity has led to a new approach in which the measurement of an intensive rather than an extensive property is the controlling factor. This method, measurement of the dielectric constant as a function of the fraction melted and correlation with the accompanying change in temperature, allows calculation of purity with high precision. The apparatus used and experimental work performed to ascertain the scope of the method are described. Determination of purity for organic compounds with differing polarizabilities and dipole moments indicate a rather wide applicability of the method. The large change in the dielectric constant of a high purity compound as it passes from solid to liquid state, with an accompanying small change in temperature, may allow automatic control of temperature within extremely narrow limits.

## 1. Cryometric Method

The well-known method of freezing point depression, (*T_f_*_0_*—T_f_*)—where *T_f_*_0_ is the freezing point of the pure material and *T_f_* that of the actual sample—provides a sensitive nondestructive measure of impurities. In this method a physical property, *ϕ*, is determined as a function of temperature, *T_x_*, in the region of the liquid-solid transition. The liquid fraction, *F*, is presumed to be a linear function of *ϕ*, that is:

$$F=\frac{\phi_{S}-\phi_{X}}{\phi_{S}-\phi_{L}}.$$ (1)

For nearly pure substances the generalized van’t Hoff equation applies:

$$\sum N_{i}=\frac{\Delta H_{f}}{RT_{f0}{\;}^{2}}.(T_{fo}-T_{x}).F$$ (2)

Σ*N_i_* designates the sum of the mole fractions of impurities soluble in the liquid and insoluble in the solid. Conventionally it is written *N*_2_*. Δ*H_f_* is the molar heat of fusion of the pure material. *R* is the gas constant. The factor ΔH*_f_*/RT*_f_*_0_^2^ is generally referred to as the cryoscopic constant, *A*, and (T*_f_*_0_—T*_x_*) is equated to Δ*T* so that eq (2) can be written:

$$1-N_{1}^{*}=N_{2}^{*}=FA\Delta T$$ (3)

Where *N*_1_* is the mole fraction of the major component.On rewriting (3) we have the straight line relationship:

$$\frac{1}{F}=\frac{A}{N_{2}^{*}}\cdot (T_{f0}-T_{x}).$$ (4)

When *F*= 1, *T_x_= T_f_*, the melting point of the sample by definition. When the line is extrapolated to $\frac{1}{F}=0$, *T_x_—T_f_*_0_, so that by thermodynamic theory applicable to dilute solutions the melting point of the pure substance can be derived experimentally from a plot of $\frac{1}{F}$ versus *T_x_.* In practice experimenters often derive *T_f_*_0_ by extrapolation of the hyperbolic relation (3) to $\frac{1}{F}=0$.

Presently there exist three widely used cryometric techniques. In dilatometry [1, 2]^1^ the sample volume is the physical property *ϕ*. The two other techniques effectively depend on heat content. They are adiabatic calorimetry [3, 4] and time-temperature (thermometric) freezing curves [5, 6]. A carefully conducted comparison [7] has shown that the three techniques, each at its best, can give reliable results. The chief advantage in the calorimetric approach is that one directly obtains accurate values for heats of fusion as well as heat capacity data for both the solid and liquid phases. The same properties can be obtained, if less accurately, from the other two techniques by auxiliary experiments.

In all cryometric techniques the maintenance of good thermal and thermodynamic equilibrium, as well as of good temperature control and measurement, are basic problems. The change in physical property, *ϕ*, associated with the liquid/solid transformation should be large in terms of measurability.

## 2. Dielectric Technique

In the dielectric technique the measured variable, *ϕ*, is the change in electrical capacitance of a capacitor with the sample as dielectric. The technique is applicable to a wide range of compounds but is subject to the following restrictions: (1) The samples must be in the purity range of 98.5 mole percent or greater; (2) the impurities must be closely related in properties to the major component; (3) the sample and impurities must have no interfacial polarization effects; (4) the sample must have high electrical resistivity. The magnitude of the change in capacitance on solid-liquid transformation depends on the polarizability, polarity, and volume changes of the sample. In general the change in capacitance of a sample during fusion is large and can be readily and accurately measured. Since the temperature variation of the dielectric constant of normal liquids and solids is quite small and since the entire melting process of nearly pure materials occurs within a small temperature range, the change in dielectric constant during the phase change (*ϕ_S_*—*ϕ_L_*) can be considered to be temperature independent. The impurities which remain in a carefully purified sample can usually be assumed to be closely related in properties to the major component. Hence, at least in the range of the static dielectric constant determination (100 to 100,000 c/s), the dielectric effects of the impurity molecules may be assumed to be negligible. Under these conditions the change in dielectric constant reflects only the change in phase whereas the temperatures depend only on the impurity.

In principle, data could be obtained from freezing or melting experiments. To approach the true equilibrium curve in a freezing experiment after the inevitable undercooling, the cell would necessarily have to be of low heat capacity. No such stringent limitation exists for apparatus designed for melting experiments. The melting process has herefore been adopted throughout.

Gravitational separation of solid during the final stages of melting may cause gross departures from uniformity in phase distribution. The method is not sensitively dependent on such uniformity but significant departures from the ideal curves occur during the last stages of melting. Glass beads are inserted to prevent the gravitational separation of the two phases.

Since the end effects in the cell are essentially constant, the measured capacitance is primarily due to that portion of the sample which is between the electrodes, and the change in capacitance is proportional to the mole fraction melted. The amount of sample above the electrode is not critical provided it represents the same composition of sample as exists between the two electrodes. However, if the cell were only partially filled, the expanding liquid would displace the air which was formerly between the electrodes, resulting in a nonlinear relationship between the capacitance change and the fraction melted.

The sample is frozen by a quenching technique so that the impurities are dispersed throughout randomly oriented small crystals. If thermodynamic equilibrium is maintained, the crystals should melt at the surfaces which are in contact with the impurities. The shape, volume and the position of the two phases change continuously during melting. Neither the Clausius-Mossotti equation nor a generalized version such as the Wiener equation can be applied. At present, it is impossible to analyze the system mathematically. However, if it is assumed that the capacitor is filled with a homogeneous dielectric whose dielectric constant is continuously changing, the experimental results here presented can be rationalized.

Equation (3) describes the hyperbolic relationship between *F* and *T_x_*, and, in analyzing the data, Saylor’s method [11] of fitting hyperbolae was used. Either the above method or the least-square fitting of the data using (4) permits calculation of *T_f_*_o_. The sample purity is calculated using eq (3) wherein *F*= 1 and *T_x_=T_f_.*

## 3. Apparatus

**Figure 1:**
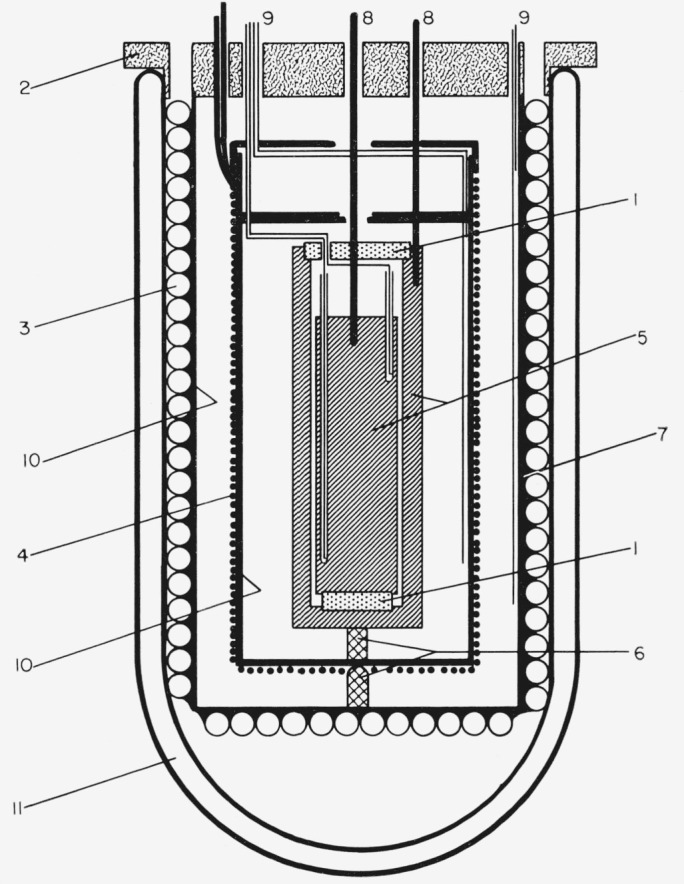
Cell and sheild assembly. 1, Fused silica 2, Lucite cover 3, Cooling coils 4, Heating wire 5, Cell walls 6, Mycalex spacers 7, Polyurethane insulation 8, Electrode leads 9, Thermocouple leads 10, Brass containers 11, Dewar Scale, 1 : 2.5

Figure 1 shows the construction of the capacitance cell and the auxiliary heating, cooling, and insulating containers. The capacitance cell is constructed of brass throughout. The 3.0 mm spacing between the inner and outer electrodes is uniformaly maintained by fused silica spacers. The capacitance, *C*_o_, of the cell in vacuum has a nominal value of 30 pf. The average temperature of the inner electrode is measured with four glass enclosed thermocouple junctions, which are sealed into holes drilled 90° apart and to different depths. A steel electrode lead is screwed into the central, high-potential electrode and a brass lead is attached to the ground-potential electrode in a similar manner. Two brass shields are used to control the cell temperature. The inner one is wrapped with asbestos-insulated Nichrome wire and the outer one has copper coils soldered to it. Each shield is fitted with a separate, four-junction series thermocouple for monitoring its temperature. The cell and the shields are insulated with carefully machined expanded polyurethane foam and are supported with Mycalex rods. The entire assembly fits in a gallon dewar and all leads protrude through the insulation, containers, and the top of the bakelite cover of the vacuum container. Electrically and thermally insulated leads, held in fixed positions throughout their lengths, are attached above the bakelite cover.

In this work both a General Radio, Type 716–C capacitance bridge and a General Radio impedance comparator have been used to measure capacitance. With the capacitance bridge, the capacitance and the dissipation factor are measured manually and directly. The field strength used is approximately 35 v/cm. The detection limits of this instrument are ±0.2 pf.

**Figure 2:**
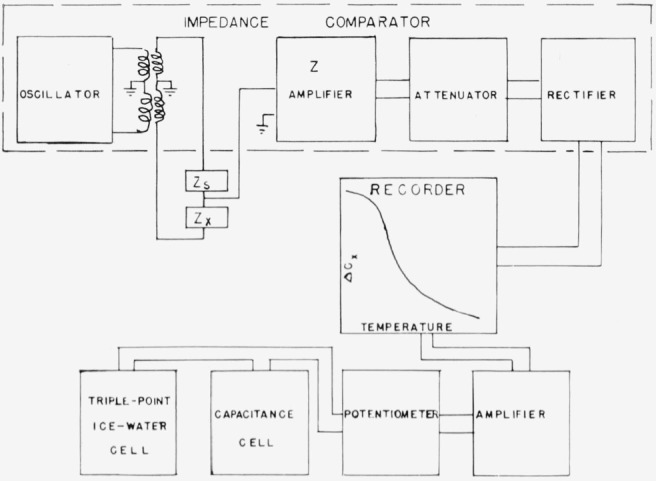
Block diagram of the measuring and recording assembly.

With the impedance comparator the capacitance of the sample can be measure using fixed frequencies of 100, 1000, 10,000, and 100,000 c/s. The impedance comparator has little or no detectable zero drift during a 24-hr period. The field strength is approximately 0.75 v/cm. A measured capacitance difference of 0.01 pf between the cell and a variable precision capacitor can be detected. The impedance comparator displays the phase angle and impedance differences independently on two meters. It also provides a direct current output which can be recorded. When the phase angle is large, it is balanced with a resistance network which has little reactance. A schematic drawing of the system used to measure and record the capacitance and the temperature of the cell is shown in figure 2. Leeds and Northrup, Speedomax, Type G, X-time and X-Y recorders are used.

An ice-water bath is used as a reference for the shield thermocouple systems, and a water-triple point cell is used as a reference for the cell system. The difference in emf between the cell thermocouples and a balancing Leeds and Northrup, Type K–3, Universal potentiometer is amplified with a Leeds and Northrup d-c microvolt indicating amplifier. The output of the amplifier is then recorded. With this system a temperature difference of 0.0001 ° C is detectable.

**Figure 3:**
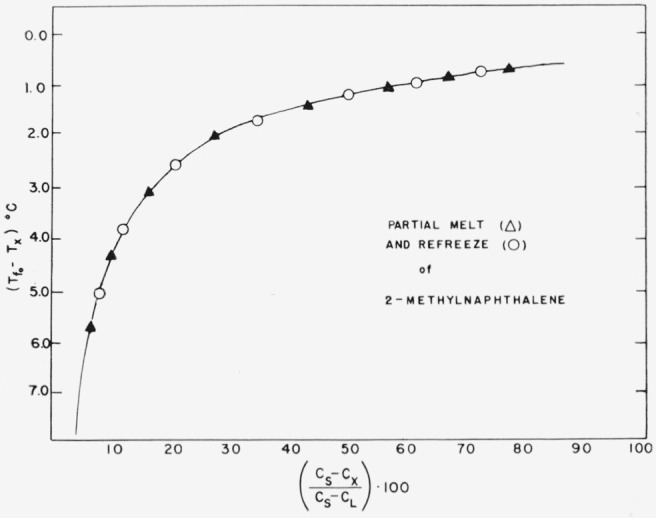
Partial melt and refreeze of 2-methylnaphthalene.

Other experiments were run to determine the minimum time necessary to insure thermodynamic equilibrium during the melting sequence. Eight hours was found to be sufficient. In still other experiments the melting process was halted and the sample was slowly refrozen. Figure 3 shows a plot of temperature versus capacitance change for one of these experiments. The fact that the two curves are superimposed illustrates one of the important advantages of the dielectric method over the thermometric method, namely that constancy in the melting rate is not necessary and that the melting sequence can even be interrupted by a period of refreezing without appreciably influencing the determination.

Freezing and melting experiments were conducted using all the fixed frequencies of the impedance comparator. No noticeable change was observed in the melting curves as a result of this change. The freezing was also done while using higher electrical field strengths, direct-current fields, and without an electrical field. In all cases the melting curves and the capacitance of the cell, when the sample was completely frozen, remained unaltered.

## 4. Experimental Detail

### 4.1 Preliminary Experiments

Series-connected, multiple-junction thermocouple systems were used in order to obtain high sensitivity and to measure temperatures which truly represented the average over the system. The uniformity of the temperature in the cell and in the first, heated shield was determined by differential temperature measurements during the melting sequence. The temperature difference between the top and bottom of both the inner electrode and the shield were recorded during preliminary melting experiments. The maximum temperature difference between the top and bottom of the shield was found to be less than 0.1 °C, and the maximum observed difference in the cell was 0.003 °C. 2-Methylnaphthalene was the material used in these experiments.

Approximate calibration of the thermocouples was achieved by immersing the sensing junctions and a calibrated platinum resistance thermometer in a well stirred oil bath whose temperature was slowly changed from 100 to 0 °C. A better calibration was achieved by performing freezing experiments with the various samples while simultaneously using both the junctions and the platinum resistance thermometer as temperature-sensing elements. As a result of this comparison, the absolute temperature of the cell, as measured by the calibrated thermocouples, is known to about 0.05 °C over the entire calibration range, and the temperature difference in the melting ranges of the samples used is accurate to the limit of the sensing equipment (0.0001 °C).

### 4.2 Experimental Determination of Purity

The liquefied sample is poured into the space between the electrodes with the top fused silica spacer removed. The amount of sample used is such that, when the sample is frozen, the solid extends just over the central electrode. After filling, the top fused silica spacer is replaced, and the cell and shields are assembled as shown in figure 1. The appropriate electrical connections are made and either a temperature-controlled liquid or gas is circulated through the coils surrounding the outer container. After the cell and its contents are cooled below the freezing point of the bulk sample, seeding is spontaneous or initiated by “sparking” the two electrode leads with a Tesla coil or by vibrating the cell. Usually freezing is very rapid, causing profuse, dendritic growth with the impurity dispersed throughout the crystalline mass. After crystallization the cell is cooled to at least 20 degrees below the melting point of the sample.

The sample is then slowly warmed by controlling the temperatures of the two shields. This slow warming allows the crystals to anneal prior to the onset of melting. The impedance of the system and the cell temperature are continuously recorded during the entire experiment. Before the onset of melting the temperature of the inner shield is maintained at no more than 0.2 °C above the cell temperature. During the melting process the temperature difference between the outer container and the cell is maintained at 0.1 °C or less. The average time required for the melting process (excluding the premelting time) is from 6 to 12 hours. The experiment is considered to be complete after the sample has been warmed 5 °C above its melting point.

Since the impedance comparator gives a linear output only when the difference between the reference and sample capacitors is no greater than 5 percent, a technique modification is needed for samples which on melting have a dielectric constant change of more than 10 percent. Nitrobenzene, for example, has a tenfold increase in dielectric constant on melting. For this substance, therefore, the temperature was recorded on an X-time recorder and the nulled value of the impedance comparator was manually recorded on the X-time strip-chart record. This required replotting to obtain the curve of 1/*F* versus *T.* With this compound the phase angle was balanced with the resistance network prior to the null-balance of the precision capacitor.

There is some experimental difficulty in the determination of the point at which the first melting takes place. Although the impedance (or capacitance) difference is the recorded variable, the impedance comparator also has a meter which shows the phase angle difference. When the sample has an appreciable polarizability or a significant dipole moment, there is a change in the phase angle prior to any noticeable change in the impedance slope. The capacitance value which corresponds to this noticeable change in phase angle is selected as the *F*= 0 value. When this is done, there is less than two percent difference between the value of (*ϕ_S_—ϕ_L_*) as determined by this method and that determined by the analysis using the hyperbolic relationship, eq (3). Such a difference causes less than one percent change in $N_{2}^{*}$.

## 5. Results of Purity Analyses

The method described has been tried for 2-methylnaphthalene, benzene, and naphthalene as examples of compounds with little polarizability and small dipole moment. In addition measurements have been obtained for *p*-dichlorobenzene, which is easily polarized but has no dipole moment. Finally nitrobenzene has been investigated as a typical substance with a large dipole moment. All compounds were distilled, dried with silica gel, and finally purified under vacuum conditions by the fractional melting technique [12]. The two samples analyzed for each compound (except 2-methylnaphthalene) represent the pure fraction and the reject fraction obtained by this technique.

**Figure 4:**
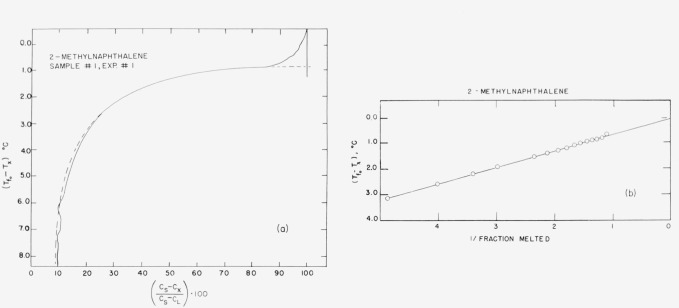
Fusion curve and 1/F presentation for 2-methylnaphthalene.

Typical experimental curves and 1/*F* versus *T* plots are shown in Figures 4 through 8. The appropriate heats of fusion were obtained from the literature references cited in the tables. Tables 1 through 6 show the results of the analyses. For comparison, one sample of each material (except 2-methylnaphthalene) was analyzed by the thermometric (time-temperature) technique.

The linear relationship between capacitance and liquid fraction (cf. sec. 1) is experimentally supported by the data here presented, because the (*C_s_—C_x_*)/(*C_s_—C*_L_)·100 versus Δ*T* graphs are hyperbolic corresponding to the generalized van’t Hoff eq (3). This linearity is further corroborated by the excellent agreement obtained between the dielectric and the time-temperature analysis.

A remaining uncertainty is due to dissolved atmospheric gases which may show differential solubilities in the liquid and the solid under test. An evacuated sealed cell would constitute an improvement over the apparatus here described. Contamination of the sample from water vapor in the atmosphere would simultaneously be eliminated. In the analyses here given for nitrobenzene and benzene the observed progressive lowering of purity may have arisen from condensed water vapor (see tables 3, 4, and 5).

The experience to date with dielectric cryoscopy is too limited for a numerical estimate of precision to be made with safety. However, it can be claimed without hesitation that this precision exceeds that presently obtainable by thermometric analysis.

## 6. Appendix. A Transfer Temperature Standard

**Figure 9:**
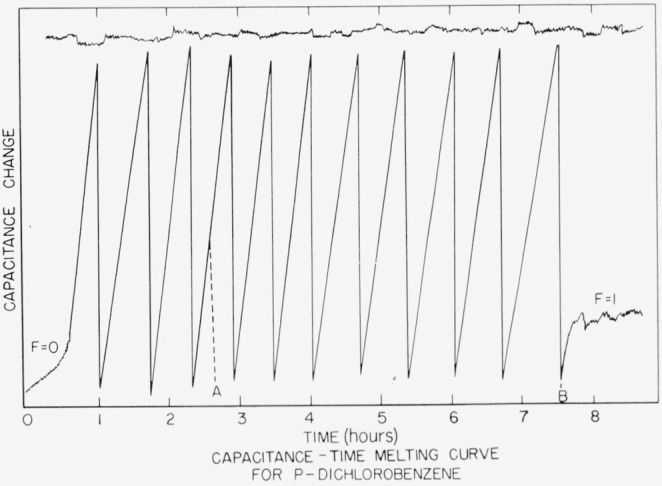
Capacitance-time melting curve for p-dichlorobenzene.

The total change in dielectric constant of any given material as it undergoes the solid to liquid phase transition is not directly related to the impurity, providing that the impurity is similar to the major component and that the measurements are conducted in the static frequency range. However, the total change in temperature accompanying such a phase change is directly proportional to the amount of impurity. If one has a thermally stable material whose total impurity content is less than ten parts per million, 90 percent of the total melting or freezing process occurs within a temperature range of approximately one or two thousandths of a degree, assuming an average cryoscopic constant. The total capacitance change of the more pure sample of *p*-dichlorobenzene as plotted on an X-time recorder is shown in figure 9. A plot of the zero stability of the impedance comparator for a similar time interval is included. The series of straight diagonal lines in the figure are a result of changing the value of the balancing precision capacitor to bring the recording on scale. From point A to point B represents a temperature change of 0.01 °C, approximately three fifths of the total melting experiment. Now, when the melting-freezing behavior of the sample is monitored by using the dielectric constant as a means of controlling the heating and cooling of the sample, a stable temperature can be obtained within the cell. When this was done manually, it was easy to maintain the dielectric constant to within 0.2 of one span of the recorded change in dielectric constant with the particular sample. When the temperature was monitored and recorded for several hours, its deviation was no greater than our ability to measure it, namely ±0.0001 °C. Because of the heavy metal construction of the present cell, overshooting of the heating-cooling cycle was unavoidable. If the monitoring of the heating and cooling cycle were automated and controlled within the same temperature limits as was done with the sample of *p*-dichlorobenzene, then direct extrapolation suggests that a cell can be designed whose temperature is stable to within 1 microdegree. In practical applications this is probably impossible, but the production of a stable, relatively sturdy cell, wherein the temperature variation could be controlled to less than 0.0001 °C, seems to be quite possible. Future plans include an attempt to redesign the cell and the shields so that a material of suitable purity can be put in an enclosed cell under its own vapor pressure to obtain transfer temperature standards which have a stability of the previously mentioned order of magnitude.

## Figures and Tables

**Figure 5:**
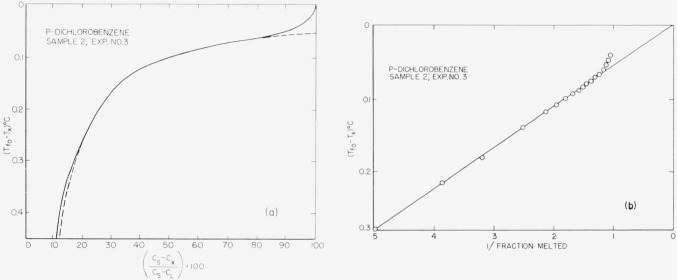
Fusion curve and 1/F presentation for p-dichlorobenzene.

**Figure 6:**
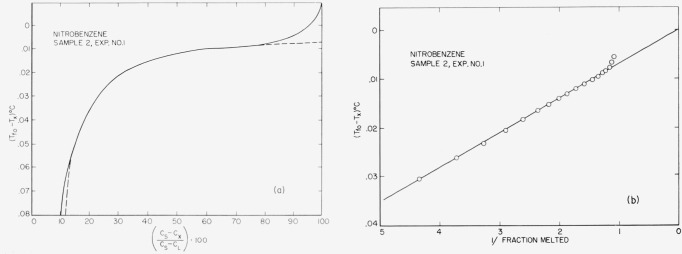
Fusion curve and 1/F presentation for nitrobenzene.

**Figure 7:**
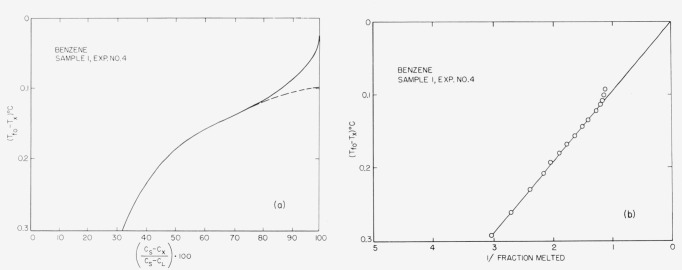
Fusion curve and 1/F presentation for benzene.

**Figure 8:**
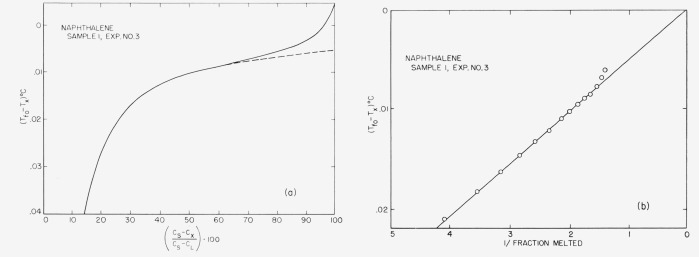
Fusion curve and 1/F presentation for naphthalene.

**Table 1:** Purity measurements on 2-methylnaphthalene

*A* =0.01541*		

Experiment	(*T_f_*_0_*–T_f_*)	Purity
		
		*Mole %*
1	0.450	99.30_7_
2	.448	99.31_0_
3	.456	99.29_7_

* The cryoscopic constant $\left(\Delta H_{f}/RT_{f0}^{2}\right)$ was obtained from data given in reference [8].

**Table 2:** Purity measurements on p-dichlorobenzene

*A* = 0.01955*			

Sample	Experiment	(*T_f_*_0_*–T_f_*)	Purity
			
			*Mole %*
1	1	0.0798	99.84_4_
	2	.0782	99.84_7_
	3	.0791	99.84_5_
	4	.0742	99.85_5_
	5	.0763	99.85_1_
	6	.0792	99.84_5_
2	1	.0492	99.90_4_
	2	.0485	99.90_5_
	3	.0497	99.90_3_
	4	.0473	99.90_7_

Thermometric measurement			

1	1		99.8_1_
	2		99.7_9_

* The cryoscopic constant $\left(\Delta H_{f}/RT_{f0}^{2}\right)$ was obtained from date given in reference [9].

**Table 3:** Purity measurements on nitrobenzene

*A* =0.01876*			

Sample	Experiment	(*T_f_*_0_*–T_f_*)	Purity
			
			*Mole* %
1	1	0.492	99.07_7_
	2	.496	99.07_0_
	3	.520	99.02_4_
	4	.525	99.01_5_

* The cryoscopic constant $\left(\Delta H_{f}/RT_{f0}^{2}\right)$ was obtained from data given in reference [10].

**Table 4:** Purity measurements on nitrobenzene^a^

*A* =0.01876*			

Sample	Experiment	(*T_f_*_0_*–T_f_*)	Purity
			
			*Mole %*
2	1	0.0126	99.976_4_
	2	.0128	99.976_0_
	3	.0134	99.974_9_
	4	.0129_5_	99.975_7_
	5	.0150	99.971_7_

Thermometric measurement			

2	1		99.95_7_
	2		99.98_9_

* The cryoscopic constant $\left(\Delta H_{f}/RT_{f0}^{2}\right)$ was obtained from data given in reference [10].

a This group of experiments was run with a glass-bead matrix between the electrodes.

**Table 5:** Purity measurements on benzene

*A* = 0.01525*			

Sample	Experiment	(*T_f_*_0_*–T_f_*)	Purity
			
			*Mole %*
1	1	0.0886	99.86_5_
	2	.0903	99.86_2_
	3	.0950	99.85_5_
	4	.0956	99. 85_4_
2	1	.0501	99. 92_3_
	2	.0537	99.91_8_
	3	.0510	99.92_2_

Thermometric measurement			

1	1		99.87
	2		99.81

* The cryoscopic constant $\left(\Delta H_{f}/RT_{f0}^{2}\right)$ was obtained from data given in reference [3].

**Table 6:** Purity measurements on naphthalen

*A*=0.01828*			

Sample	Experiment	(*T_f_*_0_*–T_f_*)	Purity
			
			*Mole %*
1	1	0.0051	99.990_61_
	2	.0052	99.990_48_
	3	.0051_7_	99.990_57_
	4	.0051_5_	99.990_59_
2	1	.0245_1_	99.95_52_
	2	.0243_7_	99.95_55_
	3	.0243_6_	99.95_50_

Thermometric measurement			

1	1		99.990
	2		99.981

* The cryoscopic constant $\left(\Delta H_{f}/RT_{f0}^{2}\right)$ was obtained from data given in reference [8].

## Footnotes

1 Figures in brackets indicate the literature references at the end of this paper.
